# Impact of spatial accessibility to primary care physicians on health care outcomes and costs

**DOI:** 10.1186/s12942-025-00430-w

**Published:** 2025-11-26

**Authors:** Yi-Xiang Weng, Ching-Chen Hsieh, Hsin-Chung Liao, Yu-Chi Tung

**Affiliations:** 1 Institute of Health Policy and Management, College of Public Health, National Taiwan University, No. 17, Xu-Zhou Road, Taipei, Taiwan; 2 Master of Public Health Program, College of Public Health, National Taiwan University, Taipei, Taiwan; 3 Department of Traditional Chinese Medicine, Chang Gung Memorial Hospital, Taoyuan, Taiwan; 4 School of Traditional Chinese Medicine, College of Medicine, Chang Gung University, Taoyuan, Taiwan; 5 National Health Insurance Administration, Ministry of Health and Welfare, Taipei, Taiwan; 6 Department of Public Administration, National Cheng-chi University, Taipei, Taiwan

**Keywords:** Primary care, Spatial accessibility, Enhanced two-step floating catchment area (E2SFCA), Geographic information systems (GIS), Health care outcomes and costs

## Abstract

**Background:**

This study is the first in Taiwan to apply the enhanced two-step floating catchment area (E2SFCA) method to evaluate the spatial accessibility of primary care. Traditional physician-to-population ratios by administrative region overlook cross-boundary healthcare-seeking and travel distance barriers. This study accounts for these limitations and further examines the impact of accessibility on healthcare utilization and outcomes.

**Methods:**

We used national health insurance claims, physician registry data, and GIS-based road networks to measure accessibility with the E2SFCA method, defining it as the number of primary care physicians per 10,000 residents within a 30-minute travel time. A retrospective cohort of 2 million adults was analyzed. Generalized estimating equations with appropriate regression models assessed associations between accessibility and healthcare utilization, expenditures, avoidable emergency department (ED), and avoidable hospitalizations.

**Results:**

Spatial analysis identified 15 townships (114,915 residents, 0.49%) with no primary care physicians and another 15 townships (114,430 residents, 0.49%) with low accessibility. These underserved areas were concentrated in central and eastern Taiwan, whereas metropolitan regions had sufficient resources. Higher accessibility was significantly associated with fewer ED visits (ratio = 0.994; 95% CI: 0.990–0.997, *P*< 0.001), ED expenditures (ratio = 0.993; 95% CI: 0.989–0.997, *P*< 0.001), the odds of avoidable ED visits (odds ratio = 0.993; 95% CI: 0.988–0.998, *P* = 0.005), and the number of avoidable ED visits (ratio = 0.993; 95% CI: 0.988–0.998, *P* = 0.004). Accessibility also reduced the odds of avoidable hospitalization (odds ratio = 0.995; 95% CI: 0.990–0.999, *P* = 0.017).

**Conclusion:**

Greater spatial accessibility to primary care was linked to reductions in ED visits, ED costs, avoidable ED use, and avoidable hospitalization. The E2SFCA method provides a more accurate tool for identifying underserved regions and can inform equitable allocation of healthcare resources. Telemedicine and mobile services should be expanded to address shortages in remote areas.

## Introduction

Accessible primary care is essential for promoting public health [1]. The World Health Organization (WHO) defines primary care as medical services characterized by first contact, accessibility, continuity, comprehensiveness, and coordinated care [2]. As a fundamental component of the healthcare system, access to primary care services is regarded as a key indicator of a country’s healthcare infrastructure, policies, and overall service quality.

Primary care typically refers to the domain of general practitioners (GPs) or primary care physicians (PCPs). In the United Kingdom, GPs account for approximately 50% of all physicians, compared to around 33% in the United States [3, 4]. The number of PCPs per 10,000 population is 9.28 in the United Kingdom, 7 in the United States, and approximately 5 in Taiwan [5, 6]. In 2024, Taiwan had a total of 55,338 physicians, of whom 20,358 (37%) were practicing in primary care clinics [7]. These figures reflect substantial variation in the structure and emphasis of primary care systems across countries.

Prior research has shown that a higher density of primary care physicians is associated with lower all-cause mortality and reduced mortality from cancer and cardiovascular diseases [8]. Longer travel distances to primary care facilities have been linked to higher stroke mortality rates [9]. At the same time, limited clinic accessibility is associated with an increased incidence of avoidable emergency department (ED) visits [10]. Improved geographic accessibility to PCPs among older adults has been shown to reduce potentially avoidable hospitalizations; notably, unbounded accessibility measures have provided stronger predictive power than bounded area-based approaches [11].

Although empirical studies have consistently demonstrated a positive relationship between primary care accessibility and health outcomes, its impact on healthcare expenditures remains less well understood. Some studies suggest that improved access to primary care reduces spending through decreases in avoidable hospitalizations and ED visits.

For instance, Gao et al. found that each additional in-person primary care visit was associated with a mean cost reduction of US $721 per patient per year, with the first visit yielding even greater savings among high-risk individuals [12]. In contrast, other studies have reported neutral or even increasing spending associated with greater primary care utilization, potentially reflecting higher use of diagnostic services, care coordination, or care management programs [13]. These inconsistencies may be due to variations in how accessibility is measured (such as provider-to-population ratios versus distance- or time-weighted metrics), differences in healthcare system structures, differences in the characteristics of study populations, and variations in the healthcare expenditure metrics used (e.g., total medical costs, outpatient vs. inpatient costs).

In recent years, the application of Geographic Information Systems (GIS) and the enhanced two-step floating catchment area (E2SFCA) method has gained traction. A growing body of research has adopted these tools to investigate the effects of spatial accessibility on healthcare costs and outcomes. This line of inquiry primarily focuses on potential spatial accessibility, commonly measured by the ratio of healthcare resources—such as medical personnel and facilities—to the population within a defined geographic distance, travel time, or transportation range [14–16]. The E2SFCA method refines accessibility assessment by incorporating travel factors such as road network distances, speed limits, and modes of transportation [14, 17–20].

In Taiwan, individuals are free to access medical services without requiring a referral from a physician. The absence of a gatekeeping or well-established primary care physician system, which allows patients unrestricted choice of providers, contributes to frequent cross-regional healthcare-seeking behavior [21–23]. Traditionally, the physician-to-population ratio has been used to assess regional healthcare accessibility [8, 24–26]; however, such metrics can be misleading in the context of frequent cross-regional utilization.

To address this limitation, this study employed the E2SFCA method to evaluate the potential spatial accessibility of primary care resources in Taiwan. Primary care clinicians were considered as the primary care resource, and their geographic distribution across villages and towns was analyzed. The objective of this study was to examine the spatial accessibility of primary care and its association with healthcare costs and outcomes, while accounting for sociodemographic and regional covariates and clustering effects at the township level. The healthcare outcomes and costs examined include the number of ED visits, hospitalizations, ED and hospitalization expenditures, total healthcare costs, the occurrence of avoidable ED visits and avoidable hospitalizations, and the frequency of avoidable ED visits and avoidable hospitalizations.

## Methods

### Data source

Demographic data, including the population of each township, city, and district, were obtained from the Ministry of the Interior’s 2017 statistical database. Data on registered physicians, hospitals, mobile physicians, and their addresses were obtained from the 2017 registry of the National Health Insurance (NHI). The NHI is a mandatory single-payer system that provides comprehensive health insurance coverage for over 99.9% of Taiwan’s population. Information on the number of primary care physicians, outpatient visits, ED services, and hospitalizations was retrieved from the 2017 and 2018 NHI claims databases. In this study, approximately 95.7% of the population had at least one recorded medical visit or pharmacy claim during the study year. Road network and distance data were acquired from the Road Network Numerical Map developed by the Transportation Research Institute under the Ministry of Transportation and Communications.

### Measurement of accessibility

This study employed the E2SFCA method, which incorporates a gravity-based weighting function to account for the effect of distance and travel time on individuals’ willingness to seek medical care [17, 19]. A key advantage of E2SFCA is its use of floating (non-administrative) boundaries, which more accurately reflect actual healthcare-seeking behavior.

Compared to the conventional two-step floating catchment area (2SFCA) method—which assumes all providers and populations within the catchment are equally accessible and does not account for distance decay —E2SFCA applies a distance decay weighting function to represent the reduced likelihood of utilizing more distant providers. Specifically, the catchment area for healthcare resources is further subdivided into subzones according to travel time. Within each subzone, the probability of utilization does not decay, but between subzones, it decreases as distance increases. Assigning different weights to different travel times overcomes the key limitation of the traditional 2SFCA method [19].

In the ***first step (supply side)***, ***supply points*** were defined as primary care clinics, with the number of physicians at each clinic geocoded accordingly. Mobile service locations, as designated and officially reported outreach sites under the NHI Medical Resource Shortage Area Improvement Program, were also included as supply points. These locations are pre-approved by the NHI and local health authorities, must operate from fixed addresses within designated resource-shortage areas, and the number of outreach physicians assigned to each location was included as appropriate. For each supply point, the total population within the designated service area was identified and weighted according to the distance decay function described above, and the provider-to-population ratio was calculated to estimate supply capacity relative to potential demand.

In the ***second step (demand side)***, ***demand points*** were defined as population locations, specifically the population-weighted centroid of each township, calculated using village-level population data. Each township’s center point was geocoded based on this weighted distribution. For each population location (e.g., township centroid), all providers within the defined catchment area were identified, and the provider-to-population ratios from the first step were summed—again weighted by distance decay—to estimate accessibility at that point.

The shortest road distance between each demand and supply point was calculated using GIS. Travel time was estimated assuming an average travel speed of 30 km/h. While prior studies in the United States have typically defined a reasonable catchment area for healthcare accessibility as a 30-minute drive or 15 miles (approximately 24 km) at an average speed of 48 km/h [27], there are substantial differences between the traffic environments of Taiwan and the United States. Taiwan generally has higher traffic density and narrower roads, resulting in slower actual travel speeds and smaller daily activity ranges. Since there is currently no universally accepted standard for travel speed in Taiwan and speed limit data are limited, we referred to previous Taiwan studies and set the average travel speed at 30 km/h for travel time calculations [28, 29]. In this study, travel time was calculated under the assumption of private vehicle (car) travel.

Based on prior research, the potential reasonable catchment area for primary care resources available to township residents was defined as a 30-minute travel distance from the township center (calculated using 30 km/h, equivalent to approximately 15 km) [10, 11, 30–32]. This area was further divided into three subzones based on travel time: 0–10 min, >10–20 min, and >20–30 min. Previous studies have suggested that appropriate distance decay parameters can be determined through expert opinion or empirical healthcare utilization data [18]. As there is currently no universal standard for distance decay rates in healthcare accessibility, we adopted a gravity-based approach following prior research [28, 29]: for each 10-minute travel interval, the probability of utilizing a healthcare resource was set to decrease by half.

For example, in the two-step calculation procedure, Step 1 (allocation of physician resources) involved identifying the population within a 30-minute travel time from township A. Because the likelihood of utilization decreases with increasing travel time, the effective population was adjusted as the sum of the population within 0–10 min, one-half of the population within 10–20 min, and one-quarter within 20–30 min. The number of physicians in township A was divided by this adjusted population to yield the provider-to-population ratio for A.

**Fig. 1:**
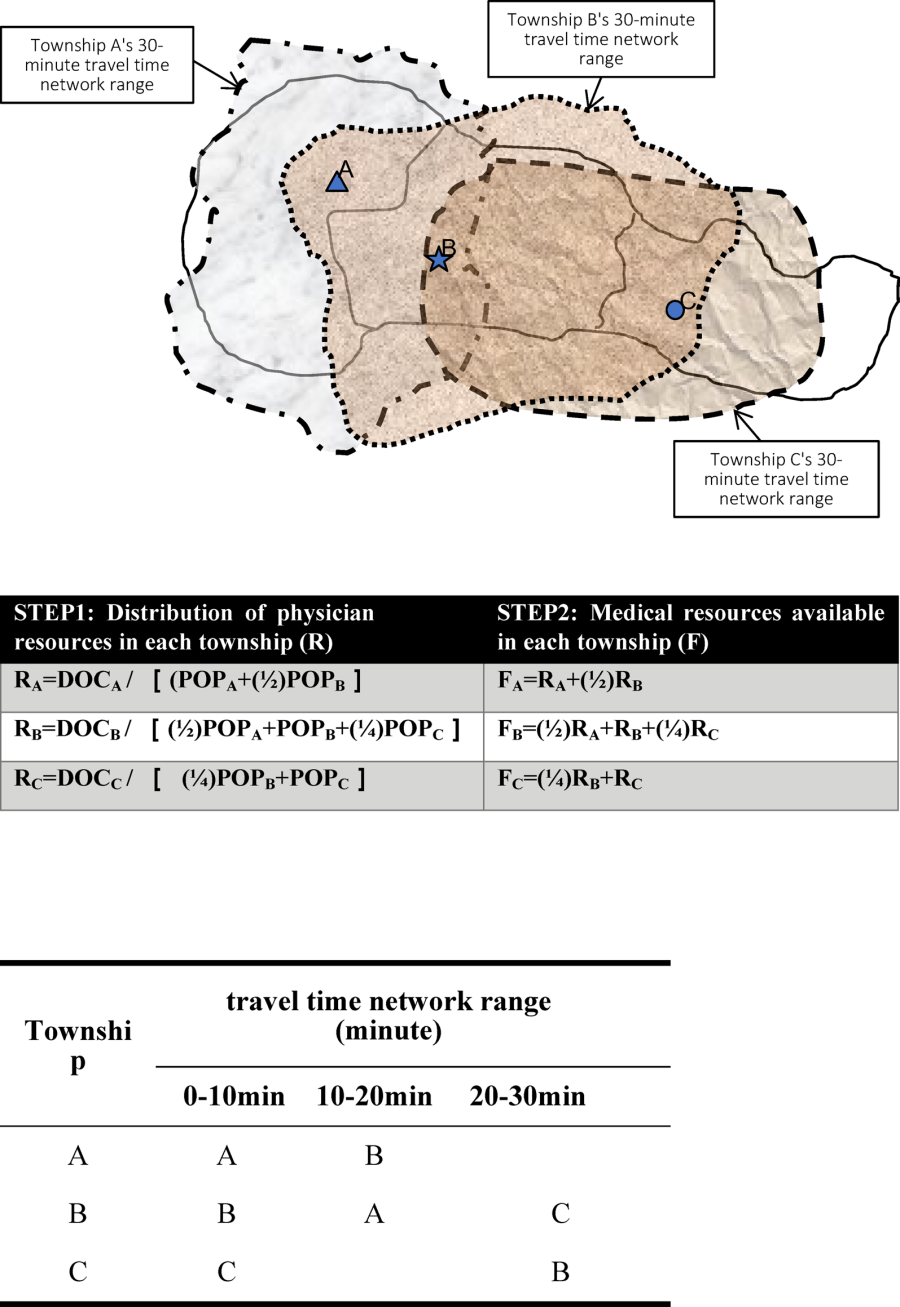
Schematic diagram of the E2SFCA method, illustrating the calculation steps and distance-decay weighting model

In Step 2 (aggregation from the demand side), the accessible physician resources for each township were also summed in a weighted manner: the sum of the provider-to-population ratios for providers within 0–10 min, one-half the sum for those within 10–20 min, and one-quarter the sum for those within 20–30 min. This approach ensures that the accessibility measure reflects the declining probability of utilizing more distant healthcare resources (Fig. 1).

### Study population

This study employed a retrospective cohort design. The initial population included 22,021,568 individuals who were enrolled in the NHI in 2017 and had at least one medical visit or pharmacy claim. Individuals aged under 18 years (*N* = 3,704,581) were excluded because healthcare utilization patterns, accessibility to care, and associated risk factors differ substantially between children and adults. In addition, children have limited autonomy in seeking healthcare, and their access is typically mediated by parents or guardians, which may bias analyses of spatial accessibility. We therefore focused on the adult population, for whom the effects of primary care accessibility are more directly relevant.

Individuals whose most frequent healthcare utilization occurred outside the main island of Taiwan (*N* = 119,140) and those with unknown sex (*N* = 2) were also excluded. After these exclusions, 18,197,845 individuals remained eligible.

**Fig. 2:**
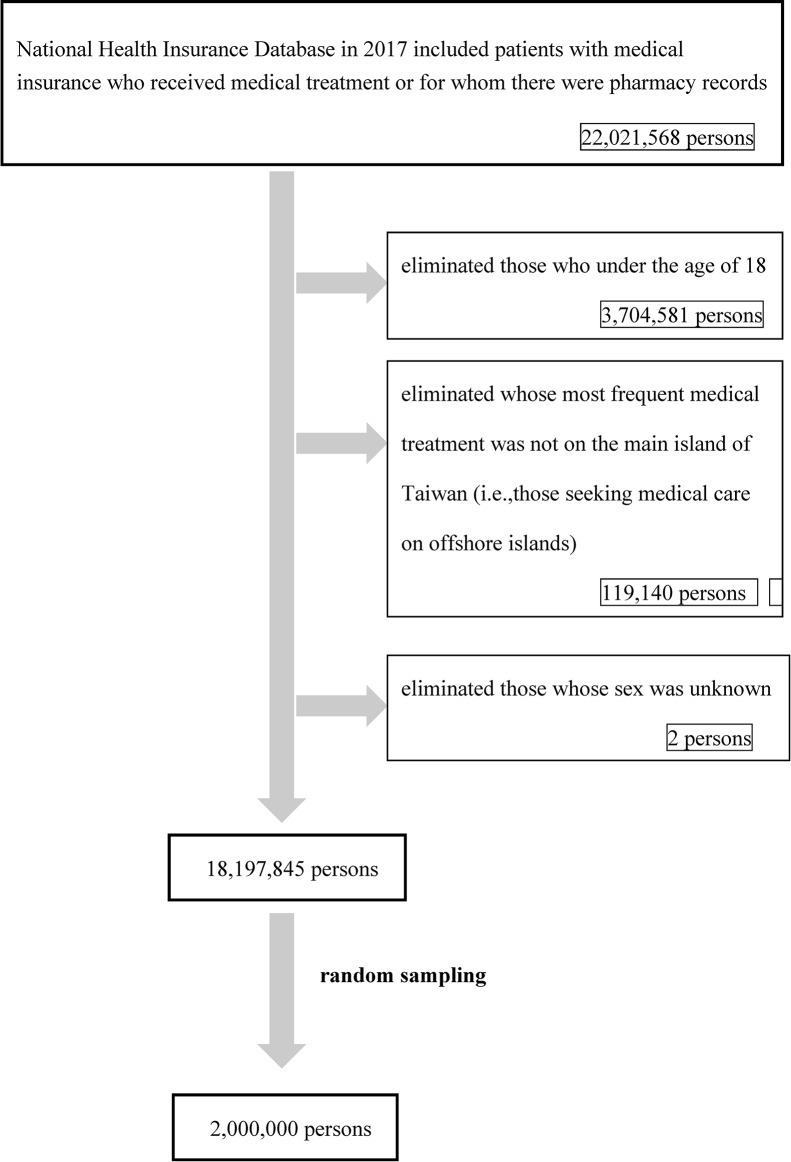
Flowchart of study subject selection from the National Health Insurance Database

Given the extremely large size of the eligible population and the associated computational burden, a simple random sample of 2,000,000 individuals was drawn for analysis (Fig. 2). To evaluate the representativeness of this sample, we compared the distributions of key characteristics—including sex, age group (categorized into three groups), and low-income status—between the sample and the full eligible population using chi-square goodness-of-fit tests. The results showed no statistically significant differences for any of these variables (all *p* > 0.05), indicating that the sample was representative of the overall population.

### Variables

The dependent variables in this study included healthcare utilization and expenditures in 2018. Specifically, the number of ED visits referred to the total ED visits per individual, and the number of hospitalizations indicated the total inpatient admissions. ED and hospitalization expenditures represented the combined total of fees paid by the NHI and patient copayments associated with these services, as recorded in the NHI claims database. Total healthcare cost was the sum of all NHI claim expenses and patient copayment for outpatient visits, ED services, pharmacy claims, and hospitalizations in 2018.

Avoidable ED visits were identified based on whether the main diagnosis matched any of the Ambulatory Care Sensitive Conditions (ACSCs) as defined by the Prevention Quality Indicators (PQIs) developed by the Agency for Healthcare Research and Quality (AHRQ) [33]. Nine PQI conditions were used: diabetes short-term complications (PQI 1), diabetes long-term complications (PQI 3), chronic obstructive pulmonary disease (COPD) or asthma in older adults (PQI 5), heart failure (PQI 8), dehydration (PQI 10), community-acquired pneumonia (PQI 11), urinary tract infection (PQI 12), uncontrolled diabetes (PQI 14), and lower-extremity amputation among patients with diabetes (PQI 16). The total number of avoidable ED visits was aggregated per individual accordingly.

Although PQIs were originally developed for evaluating avoidable hospitalizations, several published studies have applied these indicators to identify potentially avoidable ED visits, reflecting the important role of primary care in preventing acute exacerbations requiring ED care [10, 32].

Avoidable hospitalizations were similarly defined based on the principal diagnosis and aligned with PQI indicators published by AHRQ in the 2019 edition [33]. The ten selected PQIs included PQI 1, 3, 5, 7 (hypertension), 8, 11, 12, 14,15 (asthma in younger adults), and 16. The selection of these indicators followed established practices in the literature and was based on their relevance to hospitalizations that are potentially preventable through high-quality primary care [11, 25, 26]. The total number of avoidable hospitalizations per individual was computed based on these criteria.

The key independent variable was accessibility to primary care, measured using the E2SFCA method. Accessibility was defined as the number of primary care physicians per 10,000 population within a 30-minute travel radius, accounting for both supply and demand as well as spatial impedance. For each individual, accessibility was determined based on the township in which their most frequently visited outpatient clinic or pharmacy was located in 2017.

Covariates included age, sex, Charlson Comorbidity Index (CCI), low-income status, number of outpatient visits in the previous year, number of hospitalizations in the previous year, geographic location, rural residence, and township-level low-income household rate. These covariates were included to adjust for potential confounding effects on the dependent variables and to better isolate the relationship between the key independent variable (spatial accessibility) and the outcomes.

The CCI was calculated based on the weighted scoring system for 17 comorbid conditions proposed by Deyo et al. [34], and diagnosis codes were mapped using the ICD-10-CM algorithm revised by Quan et al. [35]. The higher the scores are, the greater the comorbidity burden [36–39]. Patients were then categorized into three groups according to their CCI score: 0, 1, and ≥ 2.

### Statistical analysis

ArcGIS software was used for spatial analysis and visualization. An accessibility map was generated based on the E2SFCA method, following the criteria of Taiwan’s *Improvement Program for Areas with Insufficient Medical Resources*, administered by NHI, Ministry of Health and Welfare, which defines underserved areas as those in which each physician serves more than 2,600 people [40].

All multivariable analyses were conducted under the generalized estimating equations (GEE) framework in SAS, controlling for the above-mentioned covariates and clustering at the township level. The selection of link functions was based on the type of each dependent variable: negative binomial regression with a log link was used for count outcomes (such as the number of ED visits, hospitalizations, avoidable ED visits, and avoidable hospitalizations); linear regression with an identity link was used for continuous outcomes after logarithmic transformation (such as ED expenses, hospital costs, and total costs); and logistic regression with a logit link was used for binary outcomes (including the occurrence of avoidable ED visits and avoidable hospitalizations). An exchangeable working correlation structure was specified to account for within-township correlation.

## Results

Taiwan comprises a total of 352 townships. Using the traditional physician-to-population ratio approach—which calculates accessibility based solely on the number of primary care physicians per population within administrative boundaries, without accounting for travel distance or time—2 townships were identified as having no primary care physicians, and 79 townships were classified as underserved. In contrast, 271 townships were categorized as having high levels of primary care accessibility.

According to the evaluation using the E2SFCA method, 15 townships were identified as having no accessible primary care physicians, representing a total population of 114,915 (0.49%). In addition, 15 townships with low accessibility were identified, encompassing 114,430 people (0.49%). The remaining 322 townships were classified as having higher accessibility, covering the majority of the population—23,087,473 individuals (99.02%) (Table 1).

**Table 1:** Spatial accessibility analysis of primary care resources in each Township

Access	No. township	Total popualtion (%)	Mean R	Min R	Max R	S.D R
R=0	15	114,915 (0.49%)	0	0	0	0
0<R≦3.8462	15	114,430 (0.49%)	2.12	0.18	3.72	1.22
R>3.8462	322	23,087,473 (99.02%)	19.13	4.34	79.90	9.30
Total	352	23,316,818 (100.00%)	17.59	0	79.90	10.23

R is the spatial accessibility, "accessibility" in this study is the number of primary physicians per 10,000 population within 30 minutes of travel time in each township. According to the National Health Insurance criteria for determining that the resources of physicians are insufficient -the number of physicians serving more than 2,600 (equivalent to less than 3.8462 physicians per 10,000 population), each township is divided into three categories: R=0 (accessibility zero ), 0<R≦3.8462 (insufficient accessibility) and R>3.8462 (sufficient accessibility)

**Fig. 3:**
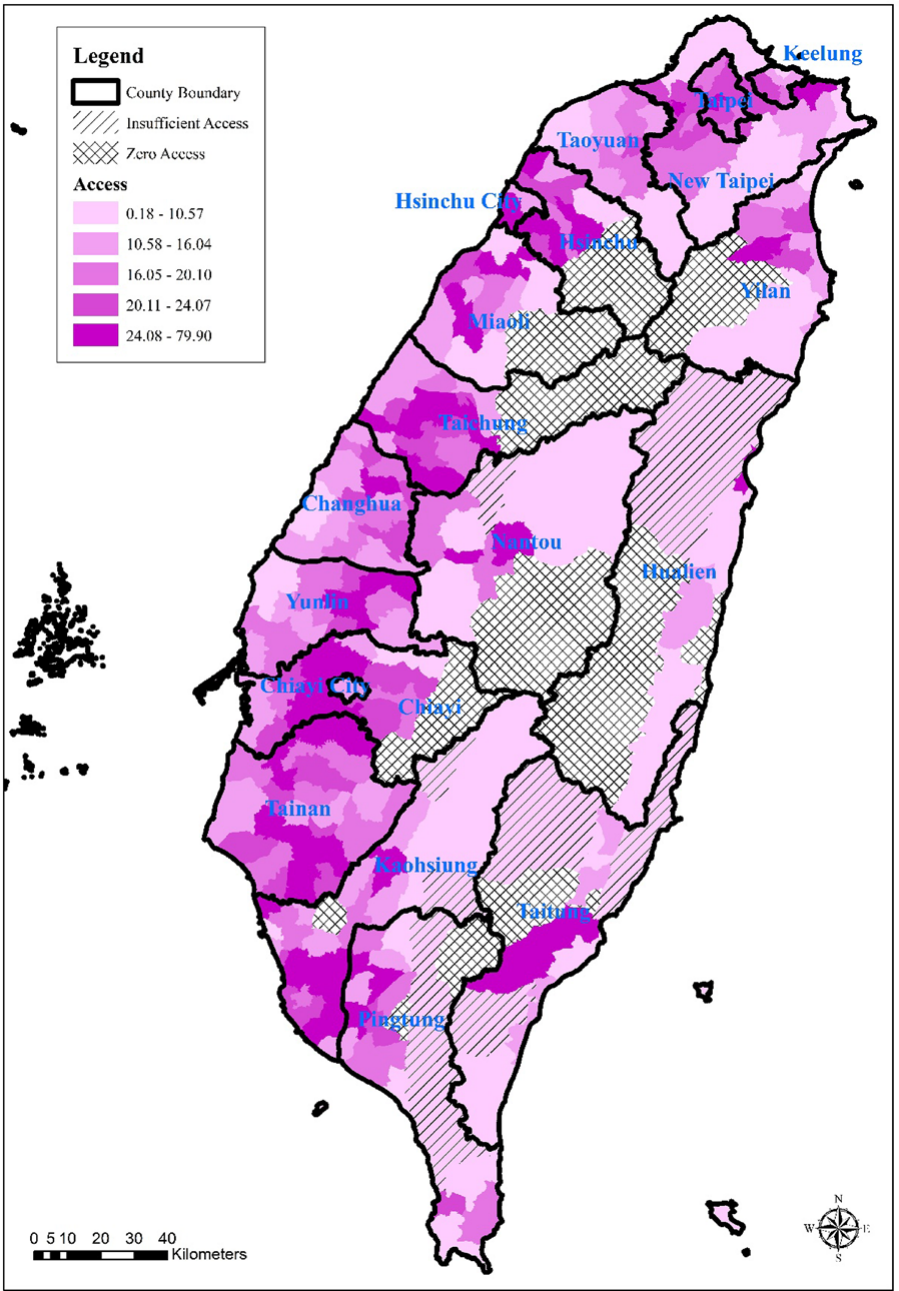
Distribution map of the spatial accessibility of primary care resources in each township

Spatial mapping revealed that townships with low primary care accessibility were predominantly located in the central and eastern regions of Taiwan’s main island, with urbanization levels classified as agricultural towns or remote rural townships. In contrast, areas with higher accessibility were mainly clustered within metropolitan urban centers. Among the 19 counties and cities in Taiwan’s main island — Keelung City, New Taipei City, Taipei City, Taoyuan City, Hsinchu County, Hsinchu City, Miaoli County, Taichung City, Changhua County, Nantou County, Yunlin County, Chiayi County, Chiayi City, Tainan City, Kaohsiung City, Pingtung County, Yilan County, Hualien County, and Taitung County — 9 had sufficient clinic-based medical resources based on accessibility thresholds (Fig. 3).

Descriptive statistics of the study variables for the 2-million-person sample are presented in Table 2. The distributions of key patient characteristics were highly consistent between the full National Health Insurance population and the sampled cohort. To further evaluate the representativeness of the sample, we conducted chi-square goodness-of-fit tests comparing sex, age group (categorized into three groups), and low-income status between the two groups. The results indicated no statistically significant differences for any of these variables (all *p* > 0.05), suggesting that the sample is representative of the overall population.

**Table 2:** Descriptive statistics of patient variables (*N* = 2,000,000)

Independent var.	*N* (Mean)	% (S.D)	Dependent var.	*N* (Mean)	% (S.D)
**Accessibility**	19.81	6.14	**No. ED**	0.28	0.99
**Age**			**No. hospitalizations**	0.15	0.66
18–49	1,136,277	56.81	**ED expenses**	1,063	5,353
50–64	513,923	25.70	**Hospital costs**	9,464	56,081
>=65	349,800	17.49	**Total costs**	31,671	109,624
**Sex**			**Avoidable ED**		
Male	949,986	47.50	No	1,947,017	97.35
Female	1,050,014	52.50	Yes	52,983	2.65
**Charlson comorbidity index**			**No. avoidable ED**	0.03	0.23
0	1,879,866	93.99	**Avoidable hospitalization**		
1	34,730	1.74	No	1,974,044	98.70
>=2	85,404	4.27	Yes	25,956	1.30
**Low-income**			**No. avoidable hospitalization**	0.02	0.20
No	1,980,791	99.04			
Yes	19,209	0.96			
**No. outpatient visits in the last year**	12.49	13.54			
**No. hospitalizations in the last year**	2.12	3.73			
**Location**					
Taipei	666,013	33.30			
North	318,958	15.95			
Central	390,221	19.51			
South	276,737	13.84			
Southernmost	305,420	15.27			
East	42,651	2.13			
**Rural residence**					
No	1,649,505	82.48			
Yes	350,495	17.52			
**Township low-income** **household rate (%)**	0.95	0.50			

After adjusting for covariates, increased accessibility to primary care was significantly associated with reduced healthcare utilization and certain outcomes (Table 3).

**Table 3:** Multivariate analysis of accessibility to dependent variables

Dependent var.	Ratio	Odds ratio	95% CI		*P* value
**No. ED**	0.994		0.990	0.997	**< 0.001**
**No. hospitalizations**	0.999		0.998	1.000	0.064
**ED expenses**	0.993		0.989	0.997	**< 0.001**
**Hospital costs**	0.999		0.998	1.000	0.228
**Total costs**	0.999		0.996	1.001	0.315
**Avoidable ED**		0.993	0.988	0.998	**0.005**
**No. avoidable ED**	0.993		0.988	0.998	**0.004**
**Avoidable hospitalization**		0.995	0.990	0.999	**0.017**
**No. avoidable hospitalization**	0.996		0.992	1.000	0.056

After controlling for age, sex, Charlson comorbidity index, low-income, outpatient visits in the last year, hospitalizations in the last year, location, rural residence, and township low-income household rate. Bold values indicate statistical significance (p < 0.05).

For every one-unit increase in accessibility, the number of ED visits decreased by 0.6% (ratio = 0.994; 95% CI: 0.990–0.997; *P* < 0.001), and the number of hospitalizations decreased by 0.1% (ratio = 0.999; 95% CI: 0.998–1.000; *P* = 0.064).

Regarding healthcare expenditures, higher accessibility was associated with significantly lower ED expenses (ratio = 0.993; 95% CI: 0.989–0.997; *P* < 0.001). Hospitalization costs (ratio = 0.999; 95% CI: 0.998–1.000; *P* = 0.228) and total medical expenditures (ratio = 0.999; 95% CI: 0.996–1.001; *P* = 0.315) were not significantly associated with accessibility.

With respect to avoidable utilization, increased accessibility was associated with a reduced odds of avoidable ED visits (odds ratio = 0.993; 95% CI: 0.988–0.998; *P* = 0.005) and a reduced number of avoidable ED visits (ratio = 0.993; 95% CI: 0.988–0.998; *P* = 0.004).

Similarly, the odds of avoidable hospitalization decreased with increasing accessibility (odds ratio = 0.995; 95% CI: 0.990–0.999; *P* = 0.017), while the reduction in the number of avoidable hospitalizations approached but did not reach statistical significance (ratio = 0.996; 95% CI: 0.992–1.000; *P* = 0.056).

## Discussion

### Statement of principal findings

Accessibility to primary care in Taiwan was generally high and was significantly associated with reductions in the risk, frequency, and cost of avoidable ED visits, as well as with a lower risk of avoidable hospitalizations.

### Interpretation in the context of the wider literature

Avoidable ED visits have been widely regarded as indicators of healthcare accessibility, the quality of primary care, and overall system performance. Greater availability of nearby primary care clinics can enable timely treatment, potentially preventing the progression of illness and thereby reducing ED service utilization. Fishman et al. reported that residents in medically underserved areas were more likely to use avoidable ED services, and while they found no significant association between physician supply and avoidable ED, clinic accessibility itself was significantly related to these outcomes [10].

The effective and timely use of primary care services has been shown to reduce avoidable ED visits. When quality outpatient care is not readily accessible, patients may seek care through the ED, increasing the likelihood of avoidable ED [30, 32]. Our findings are consistent with those of Daly et al., who demonstrated that improved geographic accessibility of primary care physicians was associated with reduced rates of potentially avoidable hospitalizations [11]. Similarly, Huang et al. reported that increased access to primary care reduced avoidable hospitalizations [30], and regional accessibility was inversely associated with avoidable hospitalization rates [25].

However, some studies have found that higher accessibility may paradoxically increase avoidable hospitalizations, potentially due to supply-induced demand [26]. Therefore, avoidable hospitalizations can serve as indirect indicators of both the accessibility and quality of primary care [25]. If accessible and timely primary care is available near the patient’s residence, the odds of avoidable hospitalizations may be reduced. Nonetheless, hospitalizations may also be driven by factors such as surgical needs or the management of complex conditions, which fall outside the scope of primary care. As such, our study found no significant associations between accessibility and total hospitalization numbers, hospitalization costs, or the frequency of avoidable hospitalizations.

### Strengths and limitations

This study employed the E2SFCA method, which incorporates the concept of activity space and a gravity model, and uses a start-end point matrix within a geographic information system to account for travel time and the decline in service utilization over distance. This approach enables a more realistic estimation of accessibility to primary care resources at the township level, overcoming limitations associated with traditional analyses based on administrative boundaries. These improvements are especially relevant in Taiwan, where cross-regional healthcare-seeking behaviors are common. By linking the number of physicians to specific clinic locations, the analysis provides a more accurate reflection of actual physician resources.

The 30-minute travel time threshold (approximately 15 km) used in this study reflects the commonly referenced definition of primary care service areas. In certain townships, clinics were absent, but hospitals were within a 30-minute range. In such cases, residents may still have access to medical care but not necessarily within a graded or referral-based system.

Several limitations should be noted. First, there is no universally accepted standard for modeling the decline rate of service use based on distance or transportation speed; this study referenced previous literature but did not differentiate between urban and rural traffic conditions. This may affect the accuracy of accessibility estimation and, consequently, its association with healthcare utilization. Second, township-level population data were obtained from the Ministry of the Interior’s Socio-Economic Database, which may not fully reflect the actual residential population. Third, the study population was restricted to individuals with medical records in the NHI Database (approximately 95.7%), which excludes individuals without healthcare utilization. Additionally, the use of population-weighted centroids may not capture actual spatial population distributions. Fourth, due to data limitations, we were unable to account for physicians’ actual working hours or the frequency of outreach medical services when estimating physician supply. Finally, this study focused on clinic-based primary care resources and did not account for hospital-based services, possibly leading to an underestimation of overall healthcare accessibility.

### Implications for policy, practice, and research

Future research should consider refining distance decay functions and travel speed assumptions based on geographic and transportation contexts. If road speed limit data or real-time traffic information becomes available, more realistic travel speeds could be applied according to different terrains and road types, or by employing services such as OSMnx for more accurate estimations, thereby enhancing the precision of accessibility modeling and the validity of study findings. Furthermore, assigning different weights to varying travel distances or times—such as applying steep versus gradual distance-decay rates—would allow for a more realistic reflection of the coverage range in the E2SFCA framework.

The integrated floating catchment area method could incorporate market competition and institutional density into accessibility measurement. Furthermore, smaller spatial units such as villages or census blocks could enable more precise identification of supply points, demand locations, and service areas, thereby enhancing the accuracy of accessibility estimations.

## Conclusions

This study demonstrated that the E2SFCA method is more effective than the traditional physician-to-population ratio in identifying townships with insufficient access to primary care services. In addition to improving measurement accuracy, the E2SFCA method provides a more realistic spatial assessment that aligns with actual healthcare-seeking behavior.

The findings highlight the potential value of the E2SFCA method as a decision-support tool for health authorities in planning the allocation of primary care resources, identifying underserved areas, and guiding targeted subsidies, workforce distribution, and infrastructure investment. Expanding the scope and eligibility of telemedicine reimbursement to rural and underserved populations would further mitigate geographic barriers to care. At the same time, clinical and primary care providers may leverage these insights to develop cross-institutional collaborations, mobile medical services, or satellite clinics to fill care gaps in remote or aging communities. Strengthening community-based health promotion and chronic disease management could also reduce avoidable hospitalizations and ED visits, thereby improving equity and efficiency in the healthcare system.

## Data Availability

The data and materials underlying the results presented in the study are available from the Taiwan National Health Insurance Administration (NHIA). Any researcher interested in accessing this dataset can submit an application to the NHIA for requesting access. Please contact the staff of the NHIA (Email: [A000322@nhi.gov.tw] for further assistance.
